# Medical students’ self-regulation of learning in a blended learning environment: a systematic scoping review

**DOI:** 10.1080/10872981.2022.2029336

**Published:** 2022-01-28

**Authors:** Rouba Ballouk, Victoria Mansour, Bronwen Dalziel, Jenny McDonald, Iman Hegazi

**Affiliations:** School of Medicine, University of Western Sydney, Sydney, Australia

**Keywords:** Medical student, self-regulated learning, blended learning, scoping review

## Abstract

**Background:**

Medical curricula are constantly evolving in response to the needs of society, accrediting bodies and developments in education and technology. The integration of blended learning modalities has challenged traditional methods of teaching, offering new prospects in the delivery of medical education. The purpose of this review is to explore how medical students adapt their learning behaviours in a Blended Learning environment to become more independent and self-regulated, in addition to highlighting potential avenues to enhance the curriculum and support student learning.

**Methods:**

Using the approach described by Levac *et al*. (2010), which builds on Arksey and O’Malley’s framework, we conducted a literature search of the following databases: MEDLINE (Ovid), ERIC, EBSCO, SCOPUS and Google Scholar, utilising key terms and variants of “medical student’, ‘self-regulated learning’ and ‘blended learning’. The search yielded 305 studies which were further charted and screened according to the Joanna Briggs Institute.

**Results:**

Forty-four studies were identified and selected for inclusion in this review. After full analysis of these studies, underpinned by Self-regulation theory, five major concepts associated with students’ learning behaviours in a Blended Learning environment were identified: Scaffolding of instructional guidance may support self-regulated learning; Self-regulated learning enhances academic performance; Self-regulated Learning improves study habits through resource selection; Blended learning drives student motivation and autonomy; and the Cognitive apprenticeship approach supports Self-regulated learning.

**Conclusion:**

This review uncovers medical students’ learning behaviours within a Blended learning environment which is important to consider for curricular adaptations and student support.

## Introduction

Medical educators continue to find more inventive and effective ways in delivering the curriculum to medical students. The delivery methods used within medical education significantly affect how students learn [1]. Although the traditional didactic lecture is still the predominant instructional method used by medical schools, it has been repeatedly criticized for being an inflexible and an ineffective way of learning that lacks student-centredness [2,3]. Blended learning involves the combination of traditional and online teaching methods to encourage a more flexible and student-centred approach to learning. Blended learning facilitates self-regulated learning and, promotes a deeper understanding of concepts, critical thinking and application of knowledge, therefore supporting a development of a fundamental learning and clinical reasoning skills [4,5].

Self-Regulated Learning (SRL) theory is useful in highlighting the strategies and barriers students experience during their interaction with various modes of delivery. SRL involves four main elements planning, learning, self-assessment, and monitoring. Zimmerman defines learners as ‘self-regulating’ when they are ‘metacognitively, motivationally, and behaviourally active participants in their own learning process’ [6]. SRL is a skill that students learn and is not inherently acquired i.e., can be learnt – and therefore taught [7]. Through guidance by the teaching faculty, a learner may be able to construct an effective Self-regulated Learning environment, a skill that is vital for continuing professional development and lifelong learning.

In a blended learning environment, a significant proportion of learning occurs outside of educational institutions. This is because blended learning offers adaptable and flexible routines for students’ learning processes [8], in contrast with the traditional learning settings where students are restricted in routine and set time schedules [5]. However, it is critical that students are able to utilise the learning opportunities that blended learning offers by practicing independent and self-directed learning [9]. For example, medical students are faced with an avalanche of resources either from within their educational institutions or externally and it is expected that students practice a level of autonomy in selecting the most appropriate resources beneficial to their learning [10]. Additionally, the requirement to acquire adult learning skills and is influenced by three important non-cognitive factors: pacing (deadlines, adhering to a schedule etc.), meaningfulness, and motivation. Therefore, it is essential that medical students have intrinsic and extrinsic motivation and planning skills for effective learning [11].

The objective of this study was to explore medical student learning behaviours, adaptations, and implementation of learning strategies in a blended learning environment using the lens of Self-Regulated Learning Theory. This insight will aid medical educators in identifying methods to enhance the curriculum and support student learning.

## Methods

We conducted a systematic scoping review to map out key findings regarding how medical students learn and self-regulate their learning in a blended learning environment by employing the five stages described originally by Arksey and O’Malley (2005) and Levac *et al*. (2010): (i) Identifying the research questions; (ii) Identifying the relevant studies; (iii) Study selection; (iv) Charting the data, and (v) Collating, summarising and reporting results [12–14].

Identifying the research questions

We sought to answer the following research questions:

1. What are the associations (if any) between Blended Learning and Self-Regulated Learning behaviours in medical students?
2. What Self-Regulated Learning strategies are used by medical students in a Blended Learning environment?
3. What Blended Learning approaches facilitate Self- Regulated Learning in medical students?

### Identifying relevant studies

A literature search was carried out, with the help of a medical librarian, between June 2019 and October 2020 with no date/year limit, searching for the main concepts in the following databases: MEDLINE (Ovid), ERIC, EBSCO, SCOPUS and google scholar. The Boolean search query used for our database search encompassed ‘medical student’ and its alternative terms, combined with the term ‘self-regulated learning’ and the varied terms of ‘blended learning’ such as e-learning, online learning and technology enabled learning.

### Study selection

**Figure 1.**
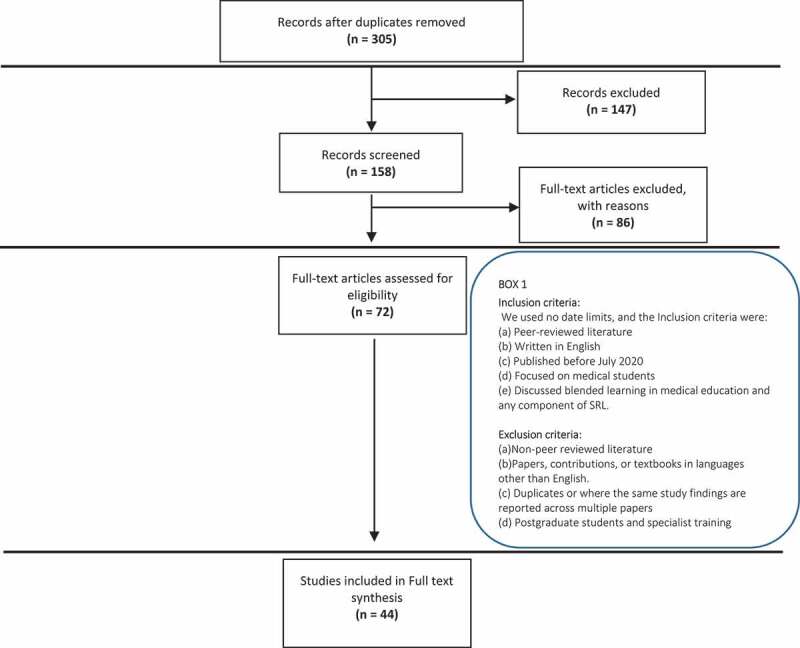
PRISMA flow diagram illustrating the different phases of the study selection process and mapping out the number of records identified based on the inclusion and exclusion criteria.

Figure 1 illustrates the search and selection process implemented by the research team, as guided by the Joanna Briggs Institute [14]. A total of 305 articles were initially identified and downloaded into EndNote®. Researchers IH, VM and RB screened article titles for abstract inclusion. Abstracts were then divided amongst the researchers IH, VM, BD and JM while RB independently screened all the abstracts (n = 158). RB met with each researcher individually to discuss their independent assessment of the abstracts to determine eligibility for full-text review against the inclusion criteria (Figure 1: Box 1). This resulted in the identification of 72 articles that were reviewed fully by all researchers. Any discrepancies were discussed amongst all researchers until consensus. Finally, 44 papers were deemed to fulfil the criteria set to be included in this review.

### Charting the data

A descriptive analysis approach was employed to the 44 articles identified from the selection process described in Figure 1. Data was recorded in an Excel spreadsheet summarising characteristics of each article including year of publication, location of study, author, research objective, type of study, data collection methods, a summary of results and key messages.

### Collating, summarising, and reporting results

The team assigned various descriptors to each full-text article that was reviewed, added to the to the Excel spreadsheet to be collated and coded by RB. RB and IH thematically analysed the findings, with reference to SRL theory, using an interpretive qualitative approach [15] to address the research questions of this study. Codes were assigned and reassigned creating categories. These categories were continuously refined evolving into conceptual themes. RB conferred the resulting themes and links to SRL with the research team (IH, JM, VM and BD), until consensus was reached.

## Results

### Descriptive summary

Of the 44 articles that we identified and analysed, 40 were published between 2015–2020 with the majority published in 2015 (n = 31) followed by those published in 2019/2020 (n = 9). The remaining 4 articles were published between 2009 and 2014. Most of the articles were comparative studies (n = 24), followed by perspective papers (n = 9), mixed methods (n = 8) and three review papers, two of which were metanalyses and one scoping. Geographic distribution revealed primary studies were conducted in the USA (n = 16), Asia (n = 11), Europe (n = 9), Australia (n = 5), Canada (n = 2) and Brazil (n = 1).

### Conceptual themes resulting from the interpretive qualitative analysis

From the 44 studies that were included in this review, we identified five conceptual themes that focused on the links between blended learning and medical student learning behaviours, in the context of SRL theory: (i) Scaffolding of instructional guidance may support self-regulated learning; (ii) Self-Regulated Learning enhances academic performance; (iii) Self-Regulated Learning improves study habits through resource selection; (iv) Blended Learning drives student motivation and autonomy; and (v) The Cognitive apprenticeship approach supports SRL. These conceptual themes are described in more detail below and are summarised in Table 1.

**Table 1.** Concepts identified from the literature that relate to medical students’ self-regulation of learning in a blended learning environment

Concepts	Links to SRL	Core points
Scaffolding ofinstructional guidance may supportSelf-Regulated Learning	Evaluation: Self-reflection. Learning to SRL	Students need more instruction in SRL and in how to monitor their learning. Insufficient direction can disadvantage students. Students who Self-identified as high achievers used monitoring strategies and proactive seeking of support more than others.
Self-Regulated Learningenhances academic performance	Monitoring and control:metacognition	There was a positive correlation between: prior knowledge and learning performance, self-efficacy and goal orientation. Self-regulation was also positively associated with learning performance.
Self-Regulated Learning improves study habits through resource selection	Behaviour: planning	There was an association between self-regulation of learning and the ability to identify relevant external resources. Self-reflection allowed students to plan what online resources they need to successfully learn and set goals for their learning.
The Cognitive apprenticeship approach supports SRL	Learning environment/context: Thinking process (cognition)	The majority of these studies discussed how a supportive environment encourages apprenticeship through strategy effective modelling. Integrating self-identity and self-regulatory behaviour through feedback, thus strengthening their skills.
BlendedLearningdrives student motivation and autonomy	Motivation: feeling and affect	Multiple studies compared two different environments, a blended classroom and a traditional one, exploring the association between autonomy and motivation. Blended classrooms prepared students more for independent and lifetime learning as it also led to an increase in engagement and motivation in comparison to didactic teaching.

#### (i) Scaffolding of instructional guidance may support self-regulated learning

Twelve studies reported on the advantages of the integration of scaffolds to support the SRL processes during medical students’ learning [4,16–26]. These studies collectively reported that enhancing students’ learning occurs through the modification and adjustment of their learning behaviour using various innovative blended teaching models and strategies, and regular feedback [19,23]. They depicted that insufficient direction and structure can disadvantage students in a blended learning classroom, especially when they are assigned extensive pre-class work [27]. Students who lacked self-regulation were less likely to review the content prior to class, limiting their ability to actively participate in the learning activity. This in turn, caused the students to lose the opportunity to engage in interactive reflective experiences to enhance and deepen their learning capabilities. However, it was found that different cohorts adopted different SRL strategies depending on their phase of learning [19,25]. SRL strategies mostly used in the early stages of learning were planning and reflection, whereas the learning and monitoring phases were less frequent. This accounts for the varying levels of SRL skill development in medical students.

#### (ii) Self-regulated learning enhances academic performance

Fourteen articles presented the relationship of SRL enhancing academic performance, whilst also examining the students’ application of deep learning strategies and the integration of knowledge [27–38]. Greater use of learning strategies, such as elaboration and critical thinking, was associated with students’ higher levels of performance in their exams [28,37]. Students adopting these learning strategies were more likely to progress and obtain better academic results than those who did not use these strategies [32].

#### (iii) Self-regulated learning improves study habits through resource selection

The association between SRL and the ability to identify relevant resources was highlighted by 9 studies that were incorporated in this review [25,29,37,39–43]. Online resources, clinical videos, and interactive resources using mobile devices were among the most relevant resources identified for use by medical students. Online resources such as quizzes and white boards provided opportunities for learner collaboration and feedback and students found that formative learning, in the form of short quizzes was a valuable tool enabling reflection and enhancing their learning processes [40]. These tools promoted their intrinsic motivation and goal-setting skills, especially when accompanied by teacher feedback. Additionally, participants using interactive resources were able to engage in more-adaptive decision-making behaviours and share their understandings when faced with rapidly evolving scenarios, more effectively than students using traditional resources [37,39]. This is also the case for researchers using hypermedia learning environments to promote and monitor SRL through learning tasks. The results confirmed that students benefited from the convenience of accessibility and more frequently accessed resources, including high-yield materials, via this medium [39,44].

#### (iv) Blended learning drives student motivation and autonomy

Eighteen studies compared the impact of different learning environments on students’ learning [4,16,26,27,33–35,40,45–54]. Having a blended learning curriculum allowed medical students to practice their SRL skills early in their medical education which led to an increase in engagement, motivation, and proactivity to learn. These skills were also shown to be essential in students’ transition into clinical training and were more successfully developed in blended learning than in a didactic teaching environment [48,55]. For example, Problem-Based Learning in a blended environment allowed students to apply SRL skills earlier than students in a traditional curriculum, increasing the former’s retention of knowledge, motivation levels and overall drive to learn [32].

#### (v) The cognitive apprenticeship approach supports SRL

The cognitive apprenticeship model encapsulated the significance of the students’ ability to learn a skill with the help of their educator [45]. Most of the studies found blended learning to be a supportive environment to encourage apprenticeship through a strategy of effective modelling and continuous feedback [17,32,35,39,56–60]. More successful students demonstrated an evolving interactive-transactive stance with their instructor, which actively strengthened their SRL skills, encouraged their participation and enhanced their professional identity development [56–58]. The processing of knowledge in fast-paced environments promoted students to foster regulation strategies and to actively adopt new skills best suited to that setting. It was evident that students’ cognitive and metacognitive skills prospered in blended learning environments whether on campus or remotely.

#### The Full Analysis of the 44 Articles Is Available in the Supplementary Materials (Table S1) [17].

## Discussion

The rapid evolution of technology has led to innovative pedagogical approaches changing the landscape of medical education. Students are now required to acquire skills from beyond traditional settings, to be successful in the more prevalent blended learning environment. On initial examination, there was limited evidence in the literature for how self-regulation can be adapted in a Blended Learning environment. This limitation of the literature informed the basis of our systematic scoping review. In this study, we aimed to analytically review the current perspectives in the literature regarding how medical students control their learning in a blended learning setting to effectively promote self-regulation of learning. We also aimed to explore and identify strategies and learning behaviours that have been shown to be effective in supporting medical students’ learning needs in a blended setting.

Blended Learning creates a medium for developing SRL skills and can be highly beneficial in preparing medical students for their continuing education after graduation [30,55]. Blended learning can be quite effective if it encompasses structures that are purposely crafted to create an environment for active participation and interaction of learners. Scaffolding of instructional guidance throughout the activity has been shown to support effective interaction within the learning process [45,56]. Studies of flexibility in adaptive scaffolding encourage customised student support until the student can independently adopt the learning process [23,61]. In a blended environment, peer learning, and help-seeking behaviours can provide external structure and guidance, varying from goal setting, strategising, planning and the development of metacognition. This will in turn accommodate students to eventually adapt to the cyclical process of SRL. However, if students are left unguided, studies have illustrated the possibility for learning to decline, emphasising the impact of direct guidance and its correlation with an increase in determination, dedication and value of accomplishing an activity [62,63].

It has been shown that a positive correlation exists between SRL and academic performance, conveying that this deep learning approach encourages learners to make sense of their studies in an active manner by using their metacognitive capabilities to self-regulate their learning [27,40]. These SRL skills are also needed for effective future clinical practice, including ongoing professional development, strategies to keep up-to-date with new medical information, and source selection especially when referring to new medical research [64]. Studies have reported that goal setting initiates the process of self-regulation and, consequently, results in higher performance for the activity at hand [64,65]. This skill is further refined through the application of tiered feedback at different stages of the process. Other studies have also acknowledged the importance of having educators trained in the application of SRL skills in a positive manner to further the academic performance of learners [66,67]. However, regardless of these developments, the evaluative measures for SRL of students in blended learning environments remains limited [68]. Furthermore, most of the SRL measures were developed for traditional settings that are still used in an online environment, finding a substantial need to develop an evaluative measure that is specific for the current and/or advancing educational settings [69].

Students conveyed that the blended classroom approach helped them to engage with content, promoted in-class participation and sustained *motivation and autonomy*. It was reported that medical students’ intrinsic motivation and autonomy, which are essential components of SRL, were facilitated by the BL environment, especially when clear student learning outcomes were incorporated into the learning activity [25,33]. Intrinsically motivated students inherit the belief of learning as challenging and rewarding at the same time. Studies have supported this finding by demonstrating that learners take initiative for their learning as they set the goals and evaluate the value of tasks to strategies [40,46].

The *cognitive apprenticeship* analogy is based on Bandura’s theory of modelling, describing the actions of humans functioning in a transaction between the self and society [70]. Hence, this model involves the reciprocal relationship between individual (medical student) influences and environmental effects (Blended learning setting). Researchers have used this model to study the core processes of the self-regulation cycle i.e., forethought, performance and reflection as educators’ direct learners’ focus for the task at hand, leading to an increase in effort and determination [63].

## Conclusion

This scoping review has identified five concepts that emphasise the association between blended learning and SRL. It exhibits that SRL mechanisms of feedback positively enhanced the understanding of medical students’ learning and academic performance. This is particularly demonstrated in a blended learning environment when sufficient guidance is provided, driving the underlying motivation of the student to learn whilst regulating their behaviour in accordance with the educator’s feedback. Feedback allows the learner to consistently reflect and adjust their learning strategies in an active manner to reach their objective. It is important to understand medical students’ study habits and how this adapts in a blended learning environment. Consequently, appropriate changes can be made to the design and delivery of the medical curriculum to support the development of SRL and enhance students’ academic abilities and future performance as practitioners. These findings will help to inform the structure, design, delivery and feedback aspects of the medical curriculum but the principles can be applicable to other disciplines.

### Limitations and future studies

The current study was constrained by the specific criteria focusing on English literature and undergraduate medical students, excluding those articles written in languages other than English, postgraduate medical students, and other health care professional education. Hence, a future focus may include expanding the scope to other disciplines, and to translate relevant non-English papers to obtain a broader insight on students’ learning behaviours in different educational settings with reference to geography and culture.

We recommend the development of an evaluative tool that measures students’ SRL strategies in a Blended Learning Setting [69]. [Ballouk R, Mansour V, Dalziel B and Hegazi I]. Through this, medical educators can gauge how students learn and how they select and use resources to support their leaning in a blended learning environment. This would help identify potential avenues to enhance and enable medical educators to better support and guide their students.

## Supplementary Material

Supplemental Material
