# Supplementary material for: Medical students’ self-regulation of learning in a blended learning environment: a systematic scoping review
Source: Med Educ Online. 2022 Jan 28;27(1):2029336. doi: 10.1080/10872981.2022.2029336 (PMC8803058; doi:10.1080/10872981.2022.2029336)
Supplement: Supplemental Material [file ZMEO_A_2029336_SM9275.docx]

| **S1: Thematic categorisation of all articles included in this scoping review.** | |  |  |  |  |
| --- | --- | --- | --- | --- | --- |
| **Title of the article** | **Authors** | **Year** | **Instrument/ Method** | **Underlying theory/Findings** | **Theme/s** |
| Problem-Based and Project-Based Learning in Engineering and Medicine: Determinants of Students' Engagement and Persistence | Bédard, Denis Lison, Christelle Dalle, Daniel Côté, Daniel Boutin, Noël | 2012 | novel survey of self-efficacy, engagement and persistence etc used to measure factors that promoted SRL in the context of PBL teaching | PBL classes in Medicine and Engineering compared; Predictor variables of engagement and persistence, with the Most potent predictor of engagement was presence of support | Blended learning drives student motivation and autonomy |
| Flipping the Advanced Cardiac Life Support Classroom with Team-based Learning: Comparison of Cognitive Testing Performance for Medical Students at the University of California, Irvine, United States | Boysen-Osborn, M. Anderson, C. L. Navarro, R. Yanuck, J. Strom, S. McCoy, C. E. Youm, J. Ypma-Wong, M. F. Langdorf, M. I. | 2016 | written test results; narrative student feedback; podcast viewing time; simulation cases and didactic content then testing: 50 multiple-choice questions | FLIP/TBL compared with lectures; A FC/TBL format for ACLS marginally improved written test results. | Self-Regulated Learning enhances academic performance |
| Determinants of Self-Regulated Learning Skills: The Roles of Tutors and Students | Demirören, Meral Turan, Sevgi Teker, Gülsen Tasdelen | 2020 | The SRL perception scales, the self-efficacy for PBL scales, tutor evaluation scale and an achievement test | SRL and self-efficacy influences effective learning in PBL; SRL associated with self-efficacy and tutor ratings; cross-sectional comparison exploring determinants of SRL including tutor and student factors in PBL context | The Cognitive apprenticeship approach supports SRL |
| A Qualitative Study of Six Medical Students in a Problem-Based Curriculum: Toward a Situated Model of Self-Regulation | Evensen, Dorothy H. Salisbury-Glennon, Jill D.    Glenn, Jerry | 2001 | central phenomenon of stance; student observations in PBL and interviews; student diaries | More successful students demonstrated an evolving interactive-transactive stance - which influenced their participation and emerging professional identity | The Cognitive apprenticeship approach supports SRL |
| Fostering Problem-Oriented Learning with Auxiliary Hypertext and Graphical Information | Fischer, Frank Grasel, Cornelia Mandl, Heinz | 2000 | through PBL; Case control study: cases presented online (haematology); pre-test: knowledge; post intervention (control and intervention groups) assessed using think aloud recording of problem solving. | Strategy modelling effective | The Cognitive apprenticeship approach supports SRL **&** Self-Regulated Learning improves study habits through resource selection |
| The Flipped Classroom and Learning Analytics in Histology | Gilliland, K. O. | 2017 | Use of learning analytics to determine engagement in a video (15 min mini-lecture). High engagement = rewinding and reviewing material repeatedly. Used the hot spots to target in BL. | Learning analytics helps identify weak areas that teachers can focus on in flipped classroom tutorials. It helps to identify topics from easy to difficult in the curriculum | Blended learning drives student motivation and autonomy |
| Flipped classroom improves student learning in health professions education: A meta-analysis | Hew, K. F. Lo, C. K. | 2018 | meta-analysis flipped vs traditional | The findings were to examine the effect of flipped classroom versus traditional classroom on student learning. More respondents reported they preferred flipped to traditional classrooms. | Blended learning drives student motivation and autonomy |
| How we developed an effective e-learning module for medical students on using professional interpreters | Umar Z Ikram 1, Marie-Louise Essink-Bot, Jeanine Suurmond | 2015 | the e-learning module: paired t-tests. Pre and post. | This e-learning module improved students' knowledge and self-efficacy in using professional interpreters during the medical interview. Thereby potentially contributing to equitable healthcare services for a linguistically diverse patient population. | Self-Regulated Learning improves study habits through resource selection **&** Self-Regulated Learning enhances academic performance |
| A randomised controlled trial of a blended learning education intervention for teaching evidence-based medicine | Dragan Ilic 1,  Rusli Bin Nordin 2, Paul Glasziou 3,  Julie K Tilson 4, Elmer Villanueva 5 | 2015 | FGDs; blended learning BL vs didactic learning DL | This study compared the effectiveness of a (BL) versus didactic (DL) approach of teaching EBM to medical students with respect to competency, self-efficacy, attitudes & behaviour toward EBM. Focus group discussions suggested a strong student preference for teaching using a BL approach, which integrates lectures, online learning and small group activities. | Blended learning drives student motivation and autonomy |
| Use of online clinical videos for clinical skills training for medical students: benefits and challenges | Hye Won Jang & Kyong-Jee Kim | 2014 | questionnaire and semi structures interviews; Discusses SRL and blended learning | The present study investigated student use and perceptions of online clinical videos for learning clinical skills and in preparing for OSCE (Objective Structured Clinical Examination). This study aims to inform us how to make more effective us of these resources. By having them on mobile devices makes them more convenient to access. | Self-Regulated Learning improves study habits through resource selection |
| Relationships between the quality of blended learning experience, self-regulated learning, and academic achievement of medical students: a path analysis | Salah Eldin Kassab 1, Ahmad I Al-Shafei 2, Abdel Halim Salem 3, Sameer Otoom 4 | 2015 | Student Course Experience Questionnaire (SCEQ), with an added e-Learning scale & self-regulated learning was measured using the (MSLQ). | Through the SCEQ scale the quality of teaching impacted motivation control of learning and intrinsic goal orientation. With The resource management regulation strategies, time and study environment, and effort regulation directly affected students' examination scores | Blended learning drives student motivation and autonomy **&** Self-Regulated Learning improves study habits through resource selection |
| Supporting Collaboration with Technology: Does Shared Cognition Lead to Co-Regulation in Medicine? | Susanne P. Lajoie  Jingyan Lu | 2012 | This is an example of SRL in different group conditions. it compares a white board with an interactive whiteboard and through the use of laptops to annotate the white board and share knowledge. Which is an example of Technology Enabled Learning. | They found that participants in the Interactive Whiteboard (IW) condition differed from the Traditional White board condition in that they engaged in more adaptive decision-making behaviour early on in the intervention. Early engagement and co-regulation occurred in the IW group which led to shared understandings and subsequently to effective patient management in latter sessions. | Self-Regulated Learning improves study habits through resource selection |
| Student perceptions of a simulation-based flipped classroom for the surgery clerkship: A mixed-methods study | Cara A. Liebert, MD  Laura Mazer, MD, MS Sylvia Bereknyei Merrell, DrPH, MS Dana T. Lin, MD James N. Lau, MD | 2016 | Medical students' perceptions of a simulation-based, the flipped classroom approach in a surgery clerkship setting. | This article investigates medical students' perceptions of a simulation-based, flipped classroom for the surgery clerkship and suggests best practices for implementation in this setting. The students reported that the learning environment fostered accountability and self-directed learning. | Blended learning drives student motivation and autonomy **&** Scaffolding of instructional guidance may support self-regulated learning. |
| The Development of Self-Regulated Learning during the Pre-Clinical Stage of Medical School: A Comparison between a Lecture-Based and a Problem-Based Curriculum | Susanna M Lucieer 1, Jos N van der Geest 2, Silvana M Elói-Santos 3, Rosa M Delbone de Faria 3 4, Laura Jonker 5, Chris Visscher 5, Remy M J P Rikers 6 7, Axel P N Themmen 8 9 | 2016 | cross sectional design (different groups at same time); self-regulation of learning self-report scale - existing validated questionnaire; quantitative | from 2nd to 6th semester there was a decline in a few SRL characteristics - both university and groups were combined; SRL between curricula no significant effect on SRL development - big effect for evaluation; LB curriculum showed decline in SRL with time i.e., sme2vs sem6 for planning, monitoring, evaluation significantly; PBL sem2vsLBsem2 only significance is with effort; PBL curricula showed increase in reflection but decline in effort between sem2 and sem6. | The Cognitive apprenticeship approach supports SRL |
| Advancing non-directive pregnancy options counseling skills: A pilot Study on the use of blended learning with an online module and simulation | Carla Lupi 1, Melissa Ward-Peterson 2, Winnie Chang 3 | 2016 | Student self-assessment & open feedback; the use of blended learning in the curriculum in the form of an online module along with a simulated exercise & its impact on student performance. explore the use of an online module to support trainee performance and explore difference in performance with & without clinical experience. | qualitative results confirm that students with prior clinical experience performed better in the GRS that assessment performance; no difference in male and female performance; quantitative results on the online e-module feedback - was useful for preparation for standardized patient exercises; module good for content coverage and approach to delivery; students gave feedback on improvements | Self-Regulated Learning enhances academic performance |
| Blended learning to teach gender in medical school | Micheal, S. Marjadi, B. | 2018 | flipped method - online content and then face to face discussions on gender & sexuality; quant and qual analyses | high response rate 90%; the flipped approach helped them increased their understanding in the topic area (pre- and post-comparison of the intervention); many features were highlighted by student that the flipped classroom approach helped them engage with constant and prompted discussions and informative; student highlighted areas for improvement to include in the constant & delivery; there was also informal feedback from a tutor perspective on impact on students for the content area | Blended learning drives student motivation and autonomy |
| Preparing Medical Students for obstetrics and gynecology milestone level one: A description of a pilot curriculum | Morgan, H. Marzano, D. Lanham, M. Stein, T. Curran, D. Hammoud, M. | 2014 | blended learning course via simulation, & then assessment; 4 week, 78 hr curriculum- theory and practical based activities and using technology for delivery; 100 mcq test pre and post course; pre and post survey on paging curriculum; skills assessment pre & post; course evaluation survey; quantitative | Knowledge had improved via post curriculum; for skills students had improved by completing the skills quicker post curriculum | Self-Regulated Learning enhances academic performance |
| Why Medical Students Choose to Use or Not to Use a Web-Based Electrocardiogram Learning Resource: Mixed Methods Study | Nilsson, M. Fors, U. Ostergren, J. Bolinder, G. Edelbring, S. | 2019 | mixed methods - learning analytics and interviews; questionnaire and exam results | neither an advantage or disadvantage to the web-based tool as it was the student’s decision to use or not | Blended learning drives student motivation and autonomy |
| Are Deep Strategic Learners Better Suited to PBL? A Preliminary Study | Papinczak, Tracey | 2009 | Quantitative and qualitative data - Medical Course Learning Questionnaire at the commencement and completion of their first year of medical studies + exam results | deep learners who are able to self-regulate adapt and appreciate the PBL process more than the surface learner who also have issues with time management | The Cognitive apprenticeship approach supports SRL **&** Self-Regulated Learning enhances academic performance |
| Effects of a Metacognitive Intervention on Students' Approaches to Learning and Self-Efficacy in a First Year Medical Course | Papinczak, Tracey Young, Louise Groves, Michele Haynes, Michele | 2008 | intervention group underwent activities of self-reflection in learning and self and peer assessment; all other students underwent PBL as normal format; questionnaire | This preliminary study employed a technique to categorise a student cohort into subgroups on the basis of their approaches to learning using the PBL tutorials. Concluding that the deep and strategic learners, appeared to be less vulnerable to the stresses of PBL in a medical course. While variation between individual learners will always be considerable. This analysis has enabled classification of a student group that may be less likely to find PBL problematic. | The Cognitive apprenticeship approach supports SRL |
| Assessing Student Engagement and Self-Regulated Learning in a Medical Gross Anatomy Course | Pizzimenti, Marc A. Axelson, Rick D. | 2015 | This study drew upon (SRL) theory to specify relevant information about learning engagement, and how the measures of particular scales might prove useful for student/faculty reflection. they tested the quality of such information as collected via the (MSLQ) via the anatomy course. | They found that students that adapted learning strategies in critical thinking and understanding had improved in their exams. These results were consistent with the course design and expectations, showing that greater use of learning strategies such as elaboration and critical thinking was associated with higher levels of performance in the course. Motivation subscales for learning were also correlated with the higher levels of performance in the course. | Blended learning drives student motivation and autonomy **&** Self-Regulated Learning enhances academic performance |
| An Interactive E-Learning Tutorial for Medical Students on How to Conduct the Performance-Oriented Mobility Assessment | Ruiz, J. G. Smith, M. Rodriguez, O. van Zuilen, M. H. Mintzer, M. J. | 2007 | e learning tutorial versus traditional; completed the iPOMA e-learning tutorial as a supplement to traditional teaching of the Performance-Oriented Mobility Assessment; pre- and post-questionnaires; quiz after tutorial and POMA assessment 1 month later | This paper describes the development, implementation, and evaluation of an interactive multimedia-based training tool designed to be integrated with traditional instructional modalities to train medical students to administer and score the POMA. They had a significant impact when students gave verbal instruction about the completion of an online tutorial. | Blended learning drives student motivation and autonomy |
| Teaching evidence-based medicine (EBM) to undergraduate medical students through flipped classroom approach | Sagheb, M. M. Amini, M. Saber, M.Moghadami, M. Nabiei, P. Khalili, R.Rezaee, R.Bazrafcan, L. Hayat, A. A. | 2018 | flipped classroom to teach EBM; student satisfaction measurement questionnaire | clarity in the design and elaboration on the results, post results were significantly better than pre. | Blended learning drives student motivation and autonomy **&** Self-Regulated Learning enhances academic performance |
| Can blended learning and the flipped classroom improve student learning and satisfaction in Saudi Arabia? | Sajid, M. R. Laheji, A. F. Abothenain, F. Salam, Y. AlJayar, D. Obeidat, A. | 2016 | Students given a questionnaire; To evaluate student academic performance and perception towards blended learning & flipped classrooms in comparison to traditional teaching. | Finding that blended learning, a relatively new concept in Saudi Arabia, shows promising results with higher student satisfaction. Flipped classrooms replace passive lecturing with active student-centered learning that enhances critical thinking & application, including information retention. | Blended learning drives student motivation and autonomy |
| The Role of Environmental and Individual Characteristics in the Development of Student Achievement: A Comparison between a Traditional and a Problem-Based-Learning Curriculum | Schauber, Stefan K. Hecht, Martin Nouns, Zineb M. Kuhlmey, Adelheid Dettmer, Susanne | 2015 | Online questionnaire | there analyses indicated that there are no substantial differences between traditional and PBL-based curricula concerning the relational structure of psychosocial variables and achievement. | The Cognitive apprenticeship approach supports SRL |
| Does blended problem-based learning make Asian medical students active learners?: a prospective comparative study | Shimizu, I. Nakazawa, H. Sato, Y. Wolfhagen, Ihap Konings, K. D. | 2019 | e-learning, i.e., ‘blended’ PBL (bPBL); seemed more effective in promoting active learning and improving knowledge, without affecting tutors’ authority. Implementing e-learning into PBL is suggested to be an effective strategy in the Asian context. | Including e- learning components in PBL such as pre-tests and post-tests improved self-efficacy and motivation in Asian students | Blended learning drives student motivation and autonomy **&** The Cognitive apprenticeship approach supports SRL **&** Self-Regulated Learning enhances academic performance |
| Assessing Medical Students' Self-Regulation as Aptitude in Computer-Based Learning | Hyuksoon S Song 1, Adina L Kalet, Jan L Plass | 2011 | They developed a Self-Regulation Measure for Computer-based learning (SRMC) tailored toward medical students, by modifying Zimmerman's Self-Regulated Learning Interview Schedule (SRLIS) for K-12 learners. | Zimmerman's SRLIS was modified (SRMC) and used medical students' SRL behaviour. SRL was found to be positively associated with academic performance | Self-Regulated Learning enhances academic performance |
| Interplay of Prior Knowledge, Self-Regulation and Motivation in Complex Multimedia Learning Environments | H.S. Song A.L. Kalet J.L. Plass | 2016 | This study examined the direct & indirect effects of medical clerkship students' prior knowledge, self‐regulation and motivation on learning performance in complex multimedia learning environments. medical clerkship students & hypermedia. | There was a positive correlation between: prior knowledge and learning performance, self-efficacy and goal orientation. Self-regulation was also positively associated with learning performance. No associations were found between learning performance and either self-efficacy or task value. | Self-Regulated Learning enhances academic performance **&** Self-Regulated Learning improves study habits through resource selection |
| Cultural Variations in E-Learning--A Case Study on Medical Training | Christina M. Steiner, Gudrun Wesiak, Adam Moore, Declan Dagger, Owen Conlan, Dietrich Albert | 2017 | Theme: behavioural section. How people learn in different ways: cultural variations in SRL | This paper presents a case study investigating the cultural dimension in a concrete e-learning application for medical training. they examined cultural variations regarding self-regulated learning. Results revealed differences in the perception of how helpful scaffolding prompts were, in the ways to express emotions, and in the acceptance of social data mining. | The Cognitive apprenticeship approach supports SRL **&** Scaffolding of instructional guidance may support self-regulated learning |
| Are the kids alright? Review books and the internet as the most common study resources for the general surgery clerkship | Janice A Taylor 1, Christiana M Shaw 2, Sanda A Tan 3, John L Falcone 4 | 2017 | a 20-item survey asking medical students about resources used in surgery | Resources used by students reflect access to high-yield material & increased Internet use. The Internet & review books were used more than the recommended textbook; Understanding study habits & resource use will help guide curricular development & students' self-regulated learning. Majority used review books and internet (Wiki). 56% never used textbooks. | Self-Regulated Learning improves study habits through resource selection |
| Framing of Feedback Impacts Student's Satisfaction, Self-Efficacy and Performance | J. M. Monica van de Ridder, Claudia M. M. Peters, Karel M. Stokking, J. Alexander de Ru & Olle Th. J. ten Cate | 2015 | it explains the importance of feedback in SRL | Performance may be enhanced by positive framing, but additional studies need to confirm this. They recommend using a positive frame when giving feedback on clinical skills. | Self-Regulated Learning enhances academic performance |
| The effectiveness of flipped classroom on learning outcomes of medical statistics in a Chinese medical school | Xiaoyu Wang Junyi Li Chengwei Wang | 2020 | Redesigning a medical statistics course, they used the MSLQ to test the SRL in flipped classroom. | As for self‐regulated learning, it significantly increased only within the flipped class, helps increase the learning outcomes, while no significant change was found within the traditional class. | Blended learning drives student motivation and autonomy **&** Self-Regulated Learning enhances academic performance |
| Smoothing out Transitions: How Pedagogy Influences Medical Students' Achievement of Self-Regulated Learning Goals | Casey B White 1 | 2007 | They used 2 different groups, two different environments, one Pbl and one traditional, they explored the links between motivation autonomy. | Basically, blended learning & in this case Pbl allowed the students to practice for SRL early on which allowed the students to transition smoothly into clerkship or clinical. thus, blended learning results in increased ability to self-regulate and independence. | Blended learning drives student motivation and autonomy |
| Creation and implementation of a flipped jigsaw activity to stimulate interest in biochemistry among medical students | Charlene Williams 1, Susan Perlis 1, John Gaughan 1, Sangita Phadtare 1 | 2018 | there are so many elements of it e.g., learner-centredness, stimulate interest (motivation), review and synthesise… | They report the creation & successful implementation of a flipped jigsaw activity that was developed to stimulate interest in learning biochemistry among medical students. The activity combined the elements of a flipped classroom for learning concepts followed by a jigsaw activity to retrieve these concepts by solving clinical cases, answering case-based questions, and creating concept maps. | Blended learning drives student motivation and autonomy |
| Self-regulated learning in a competency-based and flipped learning environment: learning strategies across achievement levels and years | Zheng, B. Ward, A. Stanulis, R. | 2019 | How do students use SRL strategies in independent learning before class? Implementation of a new competency-based curriculum followed by interviews. Qualitative research. Descriptive stats described SRL in relation to self-perceived performance. | Reporting that students use SRL strategies in different phases of learning, & how their adoption of SRL strategies differ across self-perceived achievement groups and years. Students frequently use strategies in the stages of planning and reflection, but less frequently during the learning or monitoring phase. Students who perceive themselves as high achieving, and students in their second year of medical school do use more learning strategies during the monitoring stage than their counterparts. Three constructive phases of SRL and 7 learning strategies were described. | Scaffolding of instructional guidance may support self-regulated learning |
| Factors influencing medical students' experiences and satisfaction with blended integrated e-learning | Shashidhar Venkatesh 1, Yeshwanth K Rao 2, Haleagrahara Nagaraja 1, Torres Woolley 1, Faith O Alele 1, Bunmi S Malau-Aduli 3 | 2019 | They talk about student satisfaction and experiences. We need to find out more information about their experiences relating to elements of SRL. | Wider integration of blended learning into pre-clinical undergraduate medical education could enhance the shift towards competency-based education and life-long learning among medical students. | Scaffolding of instructional guidance may support self-regulated learning |
| Advances of the innovative teaching model flipped classroom in international medical education and practice | Christopher J Ramnanan1 and Lynley D Pound2 | 2019 | Guided self-direction may support learning. | The review article about flipped classrooms, how insufficient direction and structure can disadvantage students and how students need guidance and support during the active learning sessions which is the Pre-flipped class. how flipped classrooms enhances SRL. | Scaffolding of instructional guidance may support self-regulated learning |
| Facilitating Diagnostic Competences in Higher Education--A Meta-Analysis in Medical and Teacher Education | Chernikova, Olga Heitzmann, Nicole Fink, Maximilian Christian Timothy, Venance Seidel, Tina Fischer, Frank | 2020 | collecting and integrating case-specific information to reduce uncertainty and make practical decisions, clinical reasoning to diagnose patients’ diseases accurately. | Diagnostic competences are facilitated effectively through problem-solving independent of the learners' knowledge base. Scaffolding types providing high levels of guidance are more effective for less advanced learners, whereas scaffolding types relying on high levels of self-regulation are more effective for advanced learners. | Scaffolding of instructional guidance may support self-regulated learning |
| Self-regulated learning and critical reflection in an e-learning on patient safety for third-year medical students | Gaupp, R. Fabry, G. Korner, M. | 2018 | online questionnaire the survey contained scales for SRL, reflective thinking, perceived relevance, & system usability. | identified a relationship between SRL & reflection when using an online format teaching tool; association between critical thinking and self-regulated learning; Findings show that reflection and learning skills are important factors for e-learning acceptance. | Scaffolding of instructional guidance may support self-regulated learning |
| Exploring First-Year Undergraduate Medical Students' Self-Directed Learning Readiness to Physiology | Abraham, Reem Rachel Fisher, Murray Kamath, Asha Izzati, T. Aizan Nabila, Saidatul Atikah, Nik Nur | 2011 | Self-directed learning readiness scale | Higher self-directed learning skills for high achievers. This study threw light on the fact that despite having a high desire for learning and ability of self-control, students need to be supported in their self-management skills. | Scaffolding of instructional guidance may support self-regulated learning **&** Self-Regulated Learning enhances academic performance |
| Self-regulated learning: The effect on medical student learning outcomes in a flipped classroom environment | Zheng, B. Zhang, Y. | 2020 | Quantitative study using a summarised version of the MSLQ. It looked at SRL in relation to student performance on standardised test. | The use of peer learning, and help-seeking positively affected the performance of first- and second-year students, respectively, whereas the use of rehearsal had a negative effect on student learning outcomes | Scaffolding of instructional guidance may support self-regulated learning **&** Self-Regulated Learning enhances academic performance |
| A flipped classroom method based on a small private online course in physiology | Zhang, X. M. Yu, J. Y. Yang, Y. Feng, C. P. Lyu, J. Xu, S. L. | 2019 | A comparative study. Intervention group (115) experienced a flipped classroom approach to a physiology renal block. This followed a 3-step learning process: 1. Pre-class self-study (with short pre-view videos), 2. in class knowledge internalisation, 3. After-class ability extension. Control group (82) experienced a traditional lecture-based learning approach. | The study indicated that the flipped classroom was effective in enhancing the examination scores of students, reflecting an improved learning efficiency & a deeper understanding of the knowledge. In summary, the flipped classroom based on SPOC improves learning outcomes compared with LBL and has a wide application in the learning of basic medical courses. Intervention enhanced students' exam scores and satisfaction rates. Video lecture-based preview was more effective than textbooks in preparing students for class. | Scaffolding of instructional guidance may support self-regulated learning &Self-Regulated Learning improves study habits through resource selection |
| Fostering Medical Students' Clinical Reasoning by Learning from Errors in Clinical Case Vignettes: Effects and Conditions of Additional Prompting Procedures to Foster Self-Explanations | Martin Klein 1, Bärbel Otto 2, Martin R Fischer 2, Robin Stark 3 | 2019 | error-based instructional approach; comparing two variations of online modules | The effects of the learning conditions on clinical reasoning performance were mediated by cognitive load & moderated by the students' self-efficacy which were in a web-based learning environment. Thus, improved clinical reasoning. | Blended learning drives student motivation and autonomy **&** Scaffolding of instructional guidance may support self-regulated learning |
| Self-Regulated Learning with Case-Based Worked Examples: Effects of Errors | Veronika Kopp,Robin Stark ,Nicole Heitzmann &Martin R. Fischer | 2009 | Cognitive Load, Diagnostic knowledge a case-based worked example approach was implemented in the context of a computer-based learning environment i.e., BL. | They were looking at difference between worked examples (correct and incorrect). Thus, Erroneous examples significantly increased cognitive load and decreased students’ subjective learning outcomes. | Blended learning drives student motivation and autonomy **&** Scaffolding of instructional guidance may support self-regulated learning |
|  |  |  |  |  |  |
